# Assessment of Novel Anti-thrombotic Fusion Proteins for Inhibition of Stenosis in a Porcine Model of Arteriovenous Graft

**DOI:** 10.1371/journal.pone.0137381

**Published:** 2015-09-11

**Authors:** Christi M. Terry, Ilya Zhuplatov, Yuxia He, Tze-Chein Wun, Seong-Eun Kim, Alfred K. Cheung

**Affiliations:** 1 Division of Nephrology & Hypertension, University of Utah, Salt Lake City, UT, United States of America; 2 EVAS Therapeutics, LLC, Ballwin, MO, United States of America; 3 Department of Radiology, Utah Center for Advanced Imaging Research, University of Utah, Salt Lake City, Utah, United States of America; 4 Veterans Affairs Salt Lake City Healthcare System, Salt Lake City, UT, United States of America; Kaohsiung Medical University Hospital, TAIWAN

## Abstract

**Background:**

Hemodialysis arteriovenous synthetic grafts (AVG) provide high volumetric blood flow rates shortly after surgical placement. However, stenosis often develops at the vein-graft anastomosis contributing to thrombosis and early graft failure. Two novel fusion proteins, ANV-6L15 and TAP-ANV, inhibit the tissue factor/factor VIIa coagulation complex and the factor Xa/factor Va complex, respectively. Each inhibitor domain is fused to an annexin V domain that targets the inhibitor activity to sites of vascular injury to locally inhibit thrombosis. This study’s objective was to determine if these antithrombotic proteins are safe and effective in inhibiting AVG stenosis.

**Methods:**

A bolus of either TAP-ANV or ANV-6L15 fusion protein was administered intravenously immediately prior to surgical placement of a synthetic graft between the external jugular vein and common carotid artery in a porcine model. At surgery, the vein and artery were irrigated with the anti-thrombotic fusion protein. Control animals received intravenous heparin. At 4 weeks, MRI was performed to evaluate graft patency, the pigs were then euthanized and grafts and attached vessels were explanted for histomorphometric assessment of neointimal hyperplasia at the vein-graft anastomosis. Blood was collected at surgery, immediately after surgery and at euthanasia for serum metabolic panels and coagulation chemistries.

**Results:**

No acute thrombosis occurred in the control group or in either experimental group. No abnormal serum chemistries, activated clotting times or PT, PTT values were observed after treatment in experimental or control animals. However, at the vein-graft anastomosis, there was no difference between the control and experimental groups in cross-sectional lumen areas, as measured on MRI, and no difference in hyperplasia areas as determined by histomorphometry. These results suggest that local irrigation of TAP-ANV or ANV-6L15 intra-operatively was as effective in inhibiting acute graft thrombosis as intravenous administration of heparin, but failed to inhibit hyperplasia development and stenosis in AVG.

## Introduction

Chronic hemodialysis, a life-sustaining treatment for those with end-stage renal disease, requires access to high volumetric blood flow to provide consistent shunting of blood to an extracorporeal circuit for rapid cleansing and return. Native arteriovenous fistulas, where the patient’s vein is anastomosed to the side of an artery, are the preferred means of vascular access because of low rates of infection and thrombosis. However fistulas can require several months before they can be used for hemodialysis and 20–60% never become functional [1–3]. Additionally, not all patients have blood vessels suitable for fistula creation. An arteriovenous graft (AVG), where a synthetic graft is anastomosed between an artery and a vein, can be cannulated very soon after surgical placement and can be placed in patients whose vessels are inadequate for arteriovenous fistula. Unfortunately, approximately 40 to 70% of AVG fail within one year of creation, most often due to thrombosis at the vein-graft anastomosis [1, 4, 5]. If thrombosis could be prevented or markedly delayed, the AVG would be an attractive mode for hemodialysis vascular access.

Thrombosis in AVG most commonly results from underlying neointimal hyperplasia (NH) where the blood vessel wall thickens such that the lumen narrows, restricting blood flow and promoting thrombosis [6, 7]. Aberrant hemodynamic forces, release of growth factors from activated platelets, foreign-body reactions to the AVG material, and other factors likely contribute to NH formation. Paradoxically, microthrombi can trigger the cell proliferation and migration that contributes to the NH and lumen narrowing that then promotes further thrombosis, thus perpetuating a vicious cycle [8]. During the vascular access creation surgery, the endothelial layer is injured and/or denuded. Consequently, tissue factor, the coagulation factor present on smooth muscle cell membranes, is exposed to circulating blood, initiating the extrinsic clotting cascade and thrombin generation. Thrombin cleaves fibrinogen to fibrin that polymerizes to form microthrombi that can then serve as a scaffold for smooth muscle and fibroblast cell migration and extracellular matrix deposition. Thrombin also cleaves protease-activated receptors to trigger platelet activation and release of platelet-derived growth factor that stimulates vascular smooth muscle cell proliferation [9]. Cleavage of protease-activated receptors by thrombin also triggers venoconstriction [10]. Thus thrombin generation prevents excess bleeding but also provides a fertile environment for NH development.

Systemic antithrombotic therapies have been investigated to inhibit thrombosis in AVG with some success [11] but are often associated with increased incidence of bleeding events [12, 13]. An approach that inhibits thrombin generation at the vein-graft anastomosis region may dampen the initial pro-proliferative milieu that contributes to NH formation but without increasing systemic bleeding risks. Also, previous studies have reported that local peri-operative administration of recombinant tissue factor-pathway inhibitor (TFPI) inhibited NH development after vein bypass grafting in rabbits [14], further supporting the notion that targeting thrombin generation may be an effective approach to inhibiting NH.

Two novel fusion proteins have been developed that inhibit thrombin generation in a site-directed fashion [15, 16]. Each fusion protein contains an annexin V domain that has high affinity for amino-phospholipids including phosphatidylserine and phosphatidylethanolamine. These phospholipids are typically located on the internal leaflet of cell membranes, separated from circulating blood and coagulation factors, but upon cell activation, stress, inflammation or physical injury, they are externally exposed on the lumen wall [17]. There, the amino-phospholipids promote the catalytic activities of membrane-bound tissue factor/factor VIIa, factor IXa/factor VIIIa and factor Xa/factor Va complexes and the subsequent conversion of prothrombin to thrombin [18]. The ANV-6L15 fusion protein contains the annexin V domain linked to a Kunitz protease inhibitor domain that specifically inactivates the tissue factor/factor VIIa complex while the TAP-ANV protein contains the Kunitz protease inhibitor domain that inactivates the factor Xa/factor Va prothrombinase complex. The properties of these fusion proteins make them ideal candidates to inhibit local thrombin generation and subsequent NH formation. Here we report the safety and effects of acute administration of the fusion proteins on AVG stenosis in a porcine model.

## Methods

### Synthetic arteriovenous graft (AVG) placement surgery procedure

**Fig 1:**
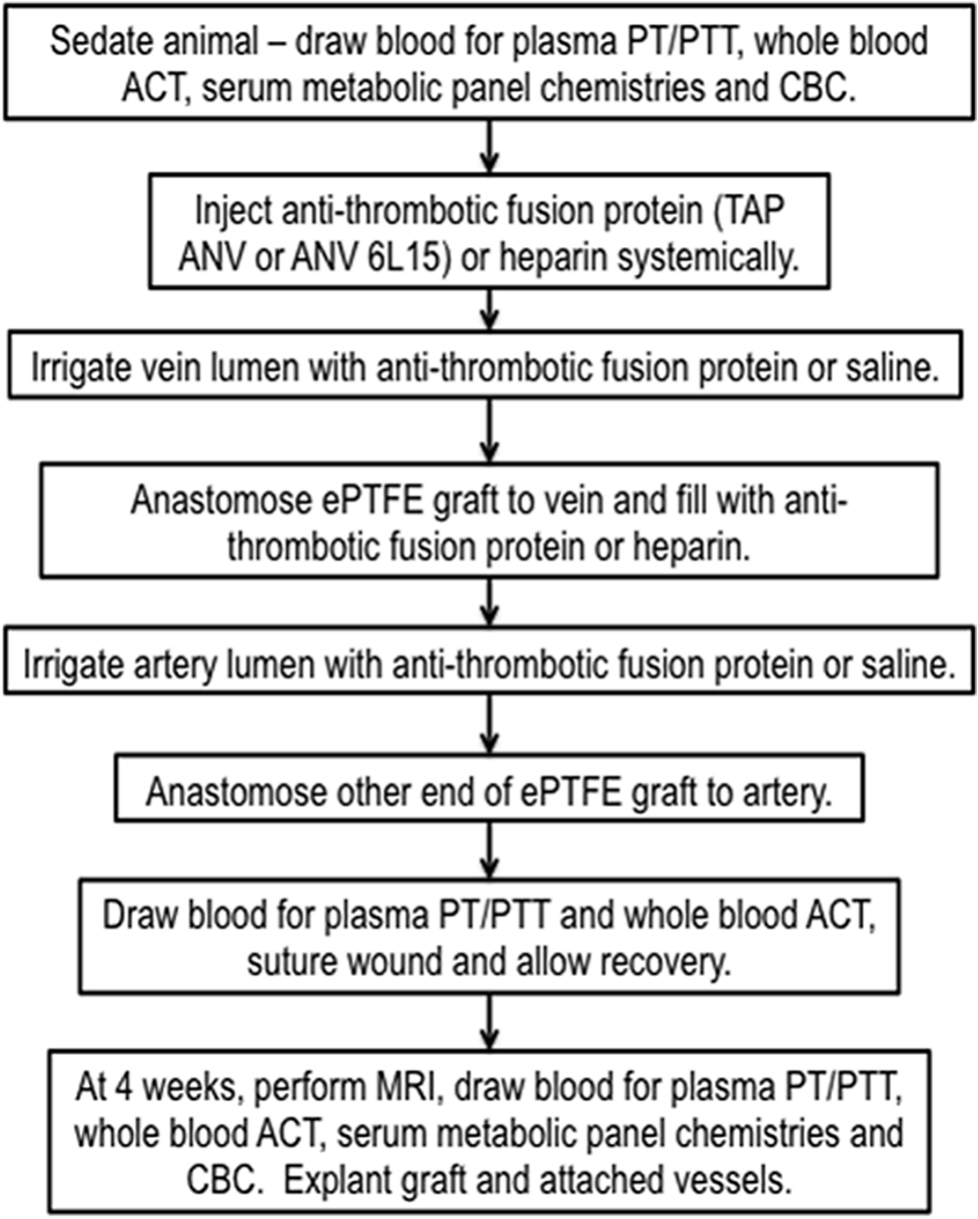
Experimental Procedure Flow Chart. PT = prothromobin time; PTT = partial thromboplastin time; ACT = activated clotting time; CBC = complete blood count.

A well-established porcine model of AVG stenosis was used for this study [19–21], which was carried out in strict accordance with the recommendations in the Guide for the Care and Use of Laboratory Animals of the National Institutes of Health. The protocol used in this study was specifically approved by the Institutional Animal Care and Use Committee of the University of Utah and this study was covered under Permit Number 12–04003. Please refer to **Fig 1**for the experimental procedure flow chart. Female Yorkshire cross domestic swine (~30kg) (Sigma Livestock, Salt Lake City, UT) were initially sedated with intramuscular injection of a Telezol® cocktail (ketamine 3–5 mg/kg (Hospira Inc., Lake Forrest, IL), tiletamine/zolazepam 3–5 mg/kg (Fort Dodge Animal Health, Fort Dodge, IA), xylaxine 6–10 mg/kg (Lloyd Laboratories, Shenandoah, IA)) and then anesthesia was maintained with isofluorane gas (1–3%) inhalation. All surgeries were performed under aseptic conditions and all efforts were made to minimize suffering. Prior to experimental treatment, peripheral blood samples were collected and used for point-of-care activated clotting times (Hemochron Response Whole Blood Coagulation System, International Technidyne Corporation, Celite activated clotting time, Edison NJ), serum metabolic panel chemistries and plasma coagulation chemistries (prothrombin times (PT) and activated partial thromboplastin times (aPTT) (Antech Diagnostics, Irvine, CA)). Antithrombotic proteins (TAP-ANV or ANV-6L15), produced as previously described [15], were diluted to the working stock concentration shortly prior to use. In sedated animals, a bolus of antithrombotic solution (3 mL at 0.1 μg/μL) was administered by intravenous injection immediately after surgical dissection of vessels. Vascular clamps were then placed on the jugular vein, proximal and distal to the site of the planned vein-graft anastomoses. A catheter was inserted into the vein lumen between the clamps and the anti-thrombotic fusion protein solution (~0.6 mL) was injected and allowed to dwell for ~3 min. A longitudinal incision (~9 mm) was then made between the clamps and a 7-cm long, 6-mm diameter, spiral-reinforced expanded polytetrafluroethylne (PTFE) graft (Bard Access Systems, Tempe AZ), cut at a 55° angle on both ends, was anastomosed to the vein by continuous suture. The graft was filled with 10 mL of the same anti-thrombotic fusion protein solution concentration that was then flushed into the jugular vein after removal of the vascular clamps. The graft was clamped and the patency and hemostasis of the vein verified. The ipsilateral artery was then similarly treated with the same anti-thrombotic fusion protein solution. The free end of the graft was then anastomosed to the artery such that the graft loop lay in the cranial direction. The clamps were removed from the artery and graft and patency and hemostasis were assessed at both anastomoses. The wound was sutured closed and peripheral blood samples were again collected for point-of-care clotting tests and plasma coagulation assays as before. Control animals were administered intravenous heparin (2000 U) and the graft was flushed with heparin (1000 U) prior to anastomoses of the free end to the artery, as per standard protocol and as described previously [20].

In all groups, treated and control, enteric-coated aspirin (81 mg/day Pharmaceutical Formulations, Edison, NJ), was administered three days before surgery and continued until euthanasia at four weeks. Clopidogrel (225 mg/day; Bristol-Myers Squibb, NY, NY) was administered one day prior to surgery and then given at 75 mg/day until euthanasia. Enrofloxacin (5 mg/kg; Bayer Corporation, Pittsburgh, PA) was administered intramuscularly on the day of surgery. A transdermal fentanyl (50 μg/pig) patch (Watson Laboratories, Inc. Corona, CA) was applied and buprenorphine (0.1 mg/kg) (Reckitt Benckiser Healthcare Ltd., Hull, England) was injected intramuscularly after surgery. Animals were allowed to recover and then monitored for untoward swelling and healing issues at the surgical site, difficulty in eating or drinking, and any signs of infection for four weeks. At four weeks, the animals were sedated, MRI was performed, peripheral blood was drawn and used for point-of-care clotting times, for serum metabolic panel chemistries and plasma coagulation chemistries. The animals were then euthanized by intravenous injection of pentobarbital sodium (80–100 mg/kg) and the graft and attached vascular tissues were obtained for histomorphological analyses.

### Activated clotting time (ACT) testing

Celite ACT tubes (ITC, Edison, NJ) were pre-warmed in the Hemochron device for 3 min prior to the addition of whole blood. The time-to-clot was determined with the Hemochron device using blood obtained immediately prior to surgery, immediately after surgery and just prior to euthanasia. The ACT was similarly measured in whole blood in which the anti-thrombotic fusion protein was added *in vitro*. For the *in vitro* studies, TAP-ANV solution was added to Celite ACT tubes prior to addition of whole blood to achieve a concentration of 40 μg/mL.

### Magnetic resonance imaging to assess AVG lumen stenosis

At 4 weeks after AVG placement, animals were sedated and placed supine in the bore of a 3 Tesla MRI machine (TIM Trio 3T, Siemens Medical Solutions, Erlangen, Germany). An overlap-decoupled 16-channel radiofrequency (RF) coil mounted on a fiberglass support molded to fit the porcine neck, or two sets of bilateral paired-array RF coils with eight total coil elements, were placed over the surgical area to obtain images. A localization scan was performed using 2D time-of-flight (TOF), with echo time (TE) of 6.08 ms and repetition time (TR) of 25 ms, and slice thickness of 3.5 mm. To visualize the graft and blood vessel lumens, T1-weighted 2D black-blood sequences with high in-plane resolution at the anastomoses were obtained using a turbo-spin echo (TSE) sequence with time-efficient double-inversion preparation (TE/TR = 9/800 ms, inversion time (TI = 500 ms, echo train length (ETL) = 9, voxel dimensions of 0.3x0.3 mm, field of view (FOV) of 192x192 mm, slice thickness of 2 mm), was applied [22]. The MR imaging was performed without contrast.

**Fig 2:**
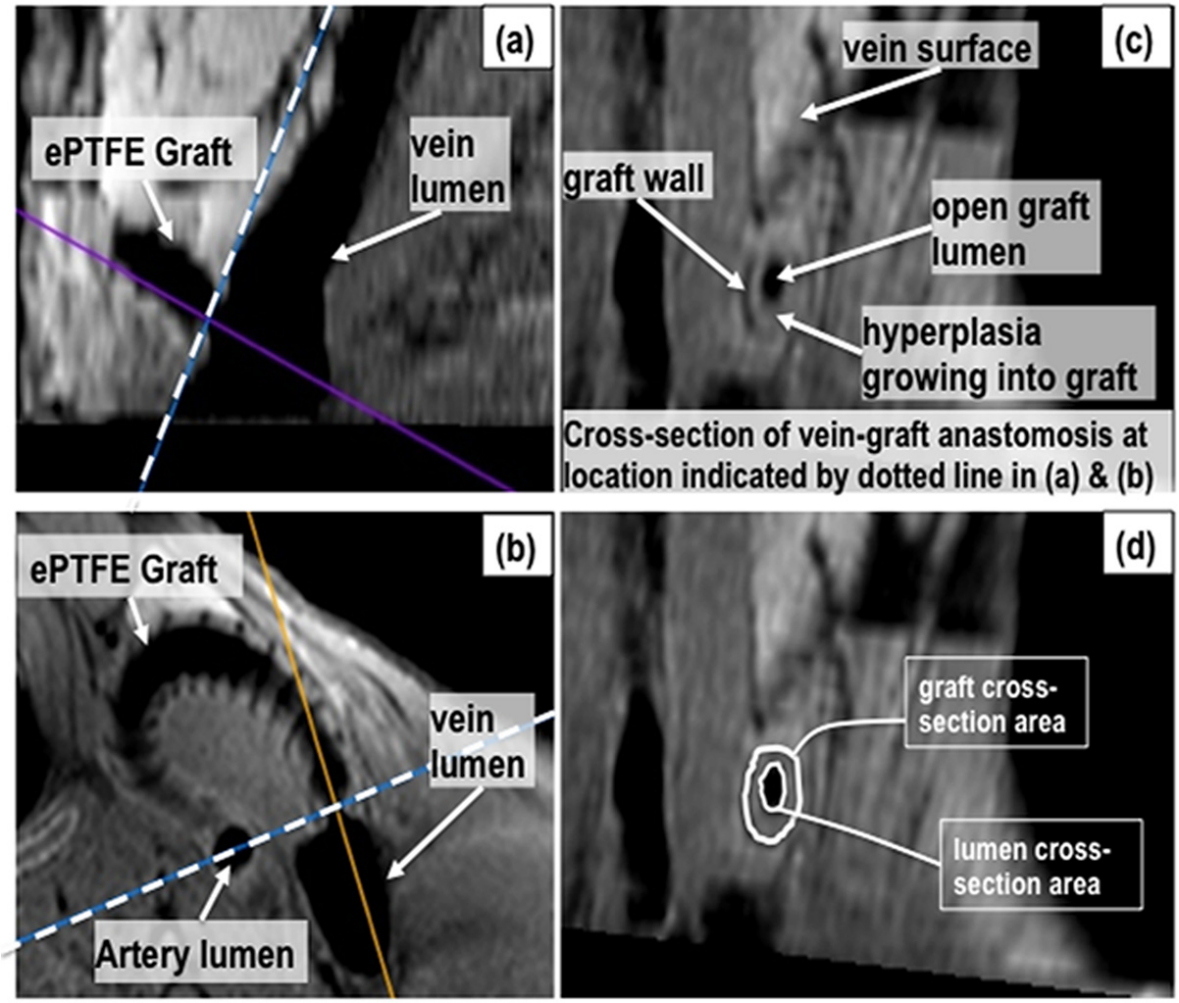
MRI analysis example. A multiplanar reconstruction of the 2D black-blood scan was performed on a pig with a graft placed between the common carotid artery and external jugular vein in the neck. The sagittal plane (a) and transverse plane (b) of the vein-graft anastomosis region were used to locate the cross-section with the smallest open lumen area at the vein-graft anastomosis (c). The graft wall and vessel lumens appear hypointense (black) in black-blood imaging. The spiral support around the graft is also hypointense and visible along the graft wall in (b). The face of the plane indicated by the dashed line in (a) and (b) is shown in (c). The lumen and graft cross-sectional areas were measured as shown in (d) using the region-of-interest pencil tool in Osirix.

OsiriX medical image processing software (Osirix v. 3.6.1), an open-source software (http://www.osirix-viewer.com/downloads.html), was used for MR image analysis. The process of image analysis has been described in detail elsewhere [21] but in brief, a 3D multiplanar reconstruction was performed using Osirix, on the 2D black-blood images. The smallest lumen cross-section area at the vein-graft anastomosis was located in the reconstruction as illustrated in **Fig 2**. The minimum lumen area was normalized by dividing by the graft cross-sectional area measured at the same location and multiplying by 100% (**Fig 2D**). This region was selected for measurement since the vein-graft anastomosis region is (i) the area in which the NH develops most frequently and to the greatest degree, and (ii) because the smallest lumen cross-sectional area (i.e., greatest stenosis) would impose the greatest limitation on blood flow. All image analyses were performed by an investigator experienced in this technique but blinded to treatment assignments.

### Histomorphometric assessment

**Fig 3:**
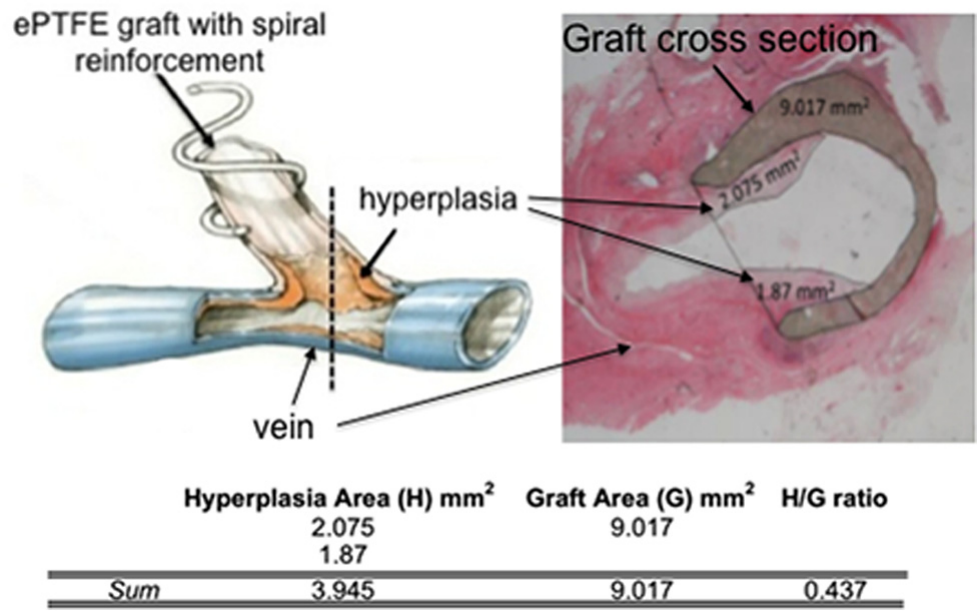
Hyperplasia-to-graft (H/G) ratio analysis example. The dashed line in the cartoon of the vein and graft on the left indicates the approximate region at the vein-graft anastomosis where the histological section, shown on the right, was collected. A line was drawn across the mouth of the graft on a digitized image of the histological section then the areas of the graft and the hyperplasia growing into the lumen of the graft were measured using NIH Image J and are shown on the histology image. The table shows the sum of the areas of the hyperplasia and the graft and then the H/G ratio results for that histological section.

At four weeks, after MR imaging, the graft and attached vessels were dissected and clamped, and *in situ* fixation was performed by filling with 10% zinc formalin prior to explantation *en bloc*. The *in situ* lumen status was also retained during the 24-h fixation in 10% zinc formalin. The tissues were fixed, paraffin embedded and stained with H&E as previously described [19, 23] to yield cross-sections of the anastomoses that contained both the graft and the native vein. An example of NH quantification at the vein-graft anastomosis is provided in **Fig 3**. All histomorphometric analyses were performed by an investigator experienced in this technique but blinded to treatment assignment.

### Statistical analysis

Differences in ACT and serum chemistries between various time points were evaluated using paired t-tests. Differences were considered significant at p ≤ 0.05. Because of skewness, paired Wilcoxon rank-sum (Mann-Whitney) tests were used to assess statistical differences among the three treatment groups for the hyperplasia-to-graft ratio (assessed by histomorphometry) and the minimal open lumen area (assessed by MRI).

## Results

Initial dose-escalation studies using 5, 20 and 100 μg/mL of the anti-thrombotic fusion proteins were performed to assess safety of the drugs before assessing efficacy. No apparent problems were observed in these initial animals so the efficacy experiments were performed using the 100 μg/mL concentration for both ANV-6L15 and TAP-ANV fusion proteins.

A total of 38 animals were used for the efficacy studies: 16 for control (heparin-treated), 12 for ANV-6L15 treatments and 10 for TAP-ANV treatments. Two were excluded due to surgical problems (1 in the control group and 1 in the ANV-6L15 group); four were excluded because of unsatisfactory histology quality (2 in the control group, 1 in the TAP-ANV group and 1 in the ANV-6L15 group). One animal was omitted from the control group due to a cardiovascular abnormality.

### Gross observations

The ANV-6L15 and TAP-ANV treatments were well-tolerated with no incidence of unusual bleeding during or after surgery similar to heparin-treated animals, and no gross difference in redness or swelling compared to heparin-treated (control) animals. No TAP-ANV- or ANV-6L15-treated animals experienced early clot formation during graft surgery, similar to the heparin-treated animals. The wound healing appeared similar and in all groups and no infections occurred in any group. One animal in the ANV-6L15 treatment group developed a large intraluminal thrombus at the vein-graft anastomosis that was detected at the 4 week MRI and which completely occluded blood flow. Upon histomorphometric analysis, the thrombus consisted mostly of fibrin and red blood cells resembling a “white clot” (data not shown).

### Blood studies

**Fig 4:**
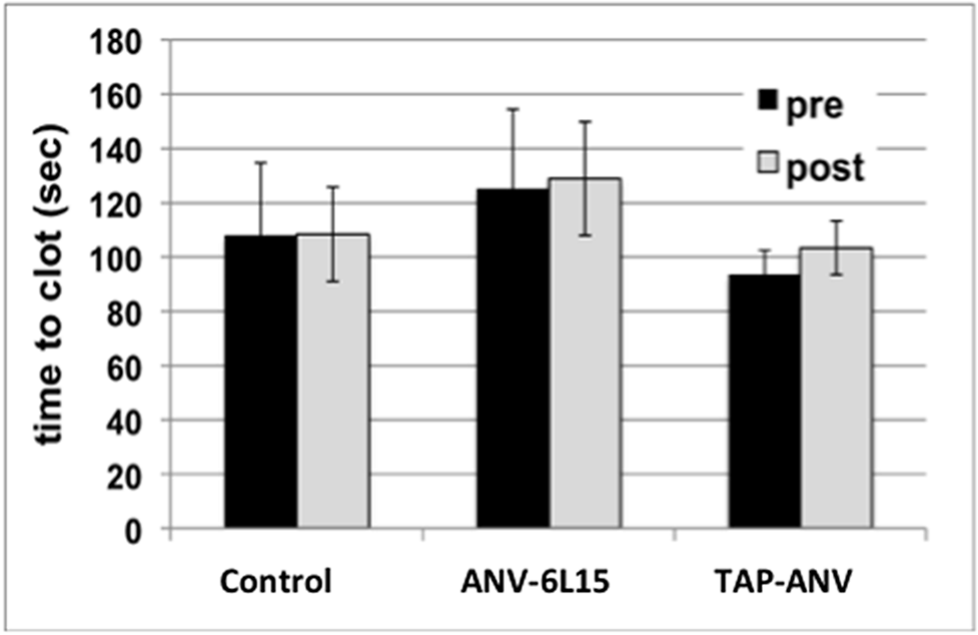
Point-of-care activated clotting times (ACT) in whole blood from heparin-treated (control), ANV-6L15-treated and TAP-ANV-treated animals. Blood was collected before (pre) and immediately after (post) AVG creation surgery. There was no significant difference between paired pre-operative and post-operative values in any group. The bars represent means ± SD.

**Fig 5:**
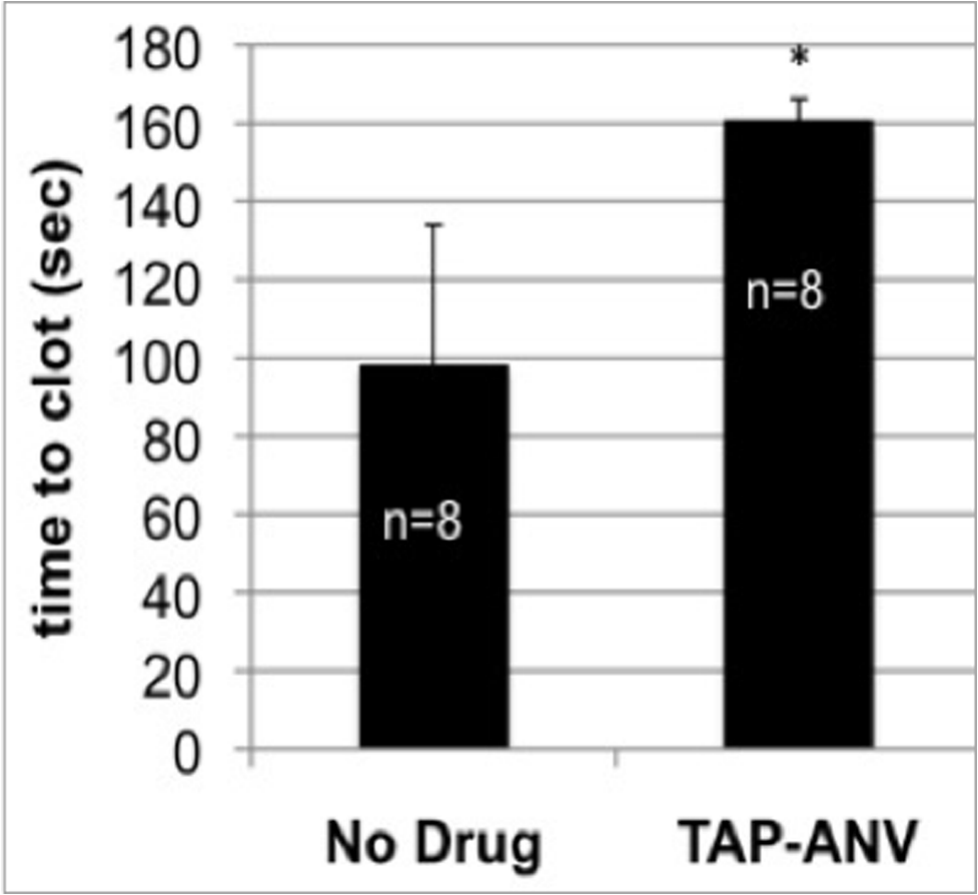
Activated clotting times (ACT) of normal porcine whole blood after the addition of TAP-ANV or with no drug. *p<0.005 when compared with no drug. The bars represent means ± SD.

Point-of-care ACT were performed on whole blood collected immediately before surgery and administration of systemic and local ANV-6L15, TAP-ANV or heparin, and immediately post-operatively, within approximately 1.5 h from the last antithrombotic drug administration. The ACT is a measure of the intrinsic coagulation pathway, with celite used as the surface activator. There was no statistically significant change in whole-blood clotting times between paired pre- and immediately post-surgery blood samples in either anti-thrombotic fusion protein-treated animals or heparin-treated animals (**Fig 4**). The activity of anti-thrombotic fusion protein was confirmed in an *in vitro* experiment in which the TAP-ANV was added directly to normal porcine whole blood and the ACT measured. As shown in **Fig 5**, the addition of TAP-ANV significantly prolonged the time-to-clot compared to the control in which no drug was added.

Heparin activates anti-thrombin that then inactivates thrombin and other proteases such as factor Xa. The antithrombotic fusion protein ANV-6L15 inactivates the tissue factor/factor VIIa complex (extrinsic pathway), and the fusion protein TAP-ANV inactivates the Xa-Va complex (common pathway). Thus, these drugs may alter the PT and aPTT. As shown in **Table 1**, in the heparin- and ANV-6L15-treated animals, the PT values from plasma collected immediately post-operatively were significantly prolonged compared to the PT values from plasma collected pre-operatively, although the values consistently remained in the normal range for swine (8.6–15.0 sec). In contrast, the PT values from plasma collected pre-operatively and post-operatively from the TAP-ANV-treated animals were unchanged. No significant difference was observed between pre-operative and immediately post-operative plasma aPTT values in any treatment group (**Table 1**). Domestic pigs are known to have hypercoagulability compared to human with much shorter average PTT values [24]. Thus it may be that higher concentrations of heparin are needed to produce significant prolongation of aPTT in swine. Also, blood was drawn approximately 1.5 h after heparin administration, which may not be the optimal time point for detecting effects. No significant differences in PT or aPTT were observed between plasma collected at 4 weeks after surgery and pre-operatively in any treatment group (data not shown).

**Table 1:** Plasma coagulation test results.

	PT (sec) (normal 8.6–15.0)			aPTT (sec) (normal 5.8–18.6)		
	*Pre	*Post	†p	*Pre	*Post	†p
**Control**	10.4 ± 0.4	10.6 ± 0.4	0.035	13.2 ± 1.9	12.8 ± 2.2	1.000
**TAP-ANV**	10.5 ± 0.7	10.8 ± 0.7	0.254	14.7 ± 5.6	13.7 ± 1.3	0.549
**ANV-6L15**	10.5 ± 0.5	10.8 ± 0.5	0.013	12.3 ± 1.4	12.6 ± 1.3	0.708

PT = prothrombin time; aPTT = activated partial thromboplastin time.

*Pre = plasma collected immediately before surgery and prior to drug administration;

Post = plasma collected immediately after surgery. Values are listed as means ± SD;

†Comparison between plasma values collected before drug administration and immediately after surgery using paired t-test. Results were considered significant at p ≤ 0.05.

Standard metabolic panel chemistries were assessed in serum collected pre-operatively and 4 weeks post-operatively. Although there were statistically significant differences between pre-operative and 4-week post-operative values for serum alanine transaminase (ALT), serum creatinine and total leukocyte counts (WBC) (**Table 2),** all values were still within normal ranges for swine. Other assessed serum chemistries were unchanged between pre-operative and 4 week post-operative time points (data not shown).

**Table 2:** Serum chemistries.

	ALT (IU/L)(normal 9–81)			Creatinine (mg/dL)(normal 1.0–3.0)			WBC count(normal 7–20 (x1000))		
	*Pre	*Post	†p	*Pre	*Post	†p	*Pre	*Post	†p
**Control**	25.9±7.2	38.2±9.6	0.001	1.0±0.2	1.2±0.2	0.001	17.4±3.5	13.6±2.5	0.002
**TAP-ANV**	22.6±8.7	32.9±7.9	0.014	1.4±0.9	1.3±0.1	0.67	15.9±5.3	13.3±2.9	0.046
**ANV-6L15**	26.0±6.4	31.1±5.9	0.029	1.0±0.1	1.1±0.2	0.020	17.4±3.1	15.6±3.9	0.371

ALT = alanine transaminase; WBC = white blood cell count.

*Pre = plasma collected immediately before surgery and prior to drug administration; Post = plasma collected immediately after surgery. Values are listed as means ± SD;

†Comparison between plasma values collected before drug administration and immediately after surgery using paired t-test. Results were considered significant at p ≤ 0.05.

### Anatomical studies

**Fig 6:**
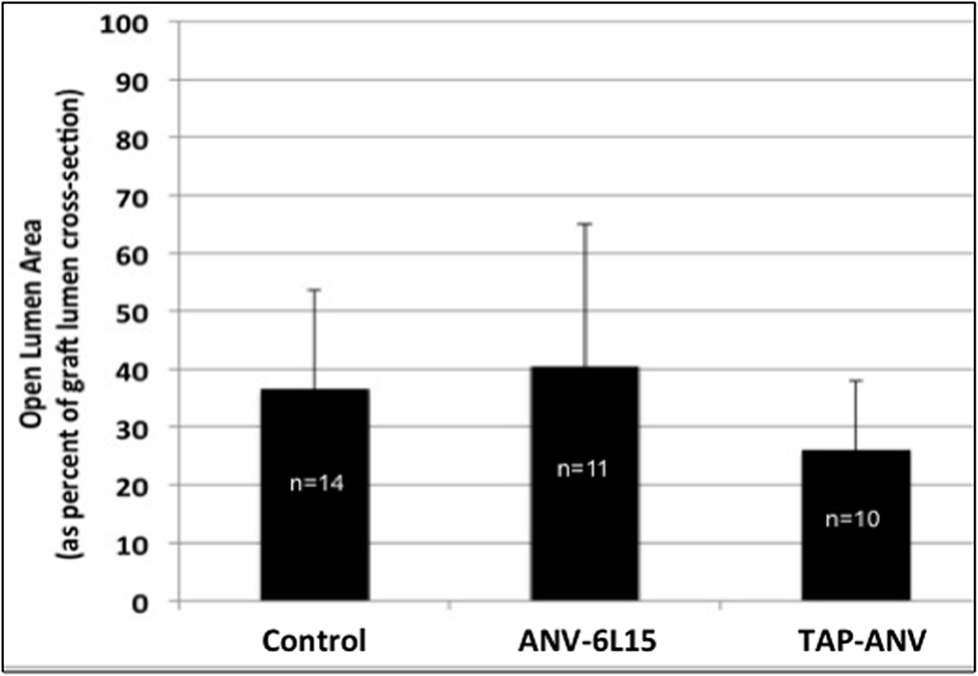
Effect of anti-thrombotic fusion protein treatment on open lumen area determined by MR imaging. Control = heparin-treated. A value of 100 would indicate the graft lumen was completely open in the vein-graft anastomosis cross-section; a value of 0 would indicate the lumen of the graft was completely occluded. There was no significant difference in the open lumen area amongst treatment groups. The bars represent means ± SD.

At 4 weeks after surgery, animals underwent MR imaging and the grafts and attached vessels were perfusion fixed and explanted for histomorphometry. MR image analysis results are shown in **Fig 6**. This analysis measured open lumen area at the narrowest region of the vein-graft anastomosis, as described previously in **Fig 2**, but cannot readily differentiate between NH and blood clot. No significant differences in mean open lumen area at the vein-graft anastomosis region were observed amongst the treatment groups.

**Fig 7:**
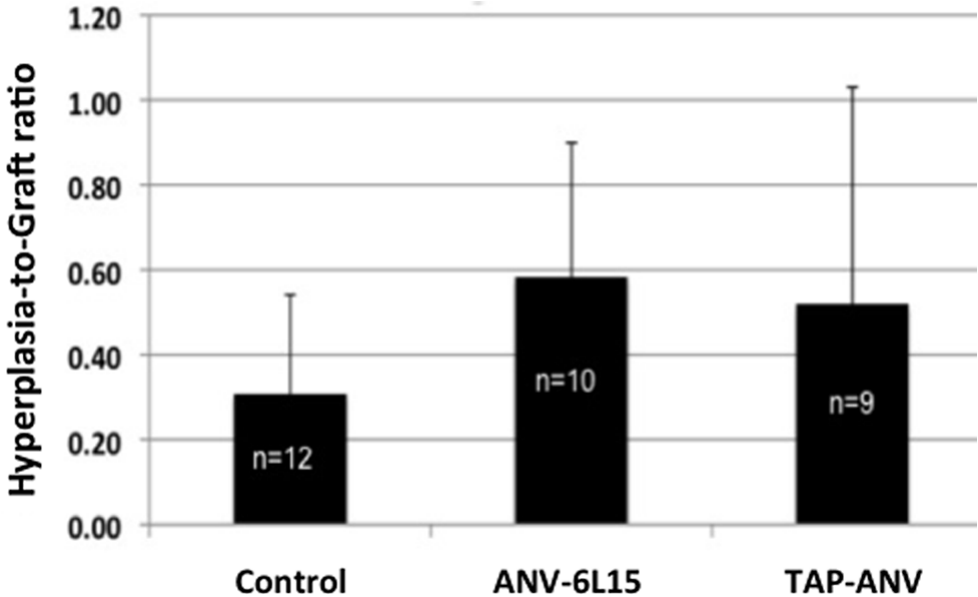
Effect of anti-thrombotic fusion protein treatment on hyperplasia-to-graft (H/G) ratios determined by histomorphometry. Control = heparin-treated. A value of 1.0 would indicate the graft was fully occluded with hyperplasia. A value of 0.0 would indicate no hyperplasia. There was no significant difference in the H/G ratio amongst the treatments groups. The bars represent means ± SD.

The NH surface area was normalized against the graft surface area in digitized images of histological cross-sections as described previously in **Fig 3**. Fresh thrombi can be differentiated from NH in this analysis. The hyperplasia-to-graft (H/G) area ratios in all three groups are shown in **Fig 7**. No significant differences in the mean H/G ratio were observed amongst the treatment groups.

## Discussion

The anti-thrombotic fusion proteins appeared to be well-tolerated, did not appear to affect wound healing or infection rates, and did not alter hemostatic parameters beyond normal ranges as assessed by point-of-care ACT assays. No peri-operative clotting events occurred in any treatment group, indicating that local treatment with the anti-thrombotic fusion proteins at the time of surgery effectively inhibited acute thrombosis similar to heparin. Significantly prolonged clotting times occurred when TAP-ANV was added directly to blood in *in vitro* analyses using the ACT assay, similar to what had been reported previously for both TAP-ANV and ANV-6L15 in tissue factor-initiated clotting assays [15]. It is expected that ANV-6L15 would also prolong the ACT but this was not tested. There was a modest yet statistically significant, acute prolongation of PT values in control and ANV-6L15-treated animals but the values were still within the normal range for swine (**Table 1**). No effect on PT or aPTT were detectable at 4 weeks after administration as expected, as the *in vivo* systemic half-life of the proteins is only hours [25]. The present data suggest that the acute local administration of the anti-thrombotic fusion proteins during surgical placement of AVG according to current protocol is safe. Nonetheless, a key finding of the present study was neither anti-thrombotic fusion protein administered in this manner inhibited NH formation.

Although some studies have reported efficacy of anti-thrombotic therapy on AVG thrombosis [26–28], a recent meta-analysis of three randomized controlled trials including a total of 956 subjects that assessed effects of systemic, prolonged, anti-platelet therapy on AVG thrombosis or patency failure reported no protection [29], suggesting systemic administration of anti-thrombotics may not deliver sufficiently high concentrations of drug to the AVG when given at doses that do not trigger adverse bleeding events. The local delivery of drug is attractive since it allows much higher targeted concentrations while minimizing the adverse effects of systemic delivery.

The failure of the peri-operative treatment with anti-thrombotic fusion proteins to suppress NH formation and stenosis in the present porcine AVG study stands in sharp contrast with previous studies in other animal models that involved simpler forms of vascular injury, such as balloon angioplasty and interposition vein grafting. Tissue factor pathway inhibitor (TFPI) protein is a Kunitz-type protease inhibitor of the coagulation cascade. Local irrigation with recombinant TFPI or the TFPI gene has been shown in rabbit models to inhibit NH formation in vein bypass grafts [14] and after arterial angioplasty [30–32]. Three-hour or 24-h intravenous infusion of hirudin (an inhibitor of thrombin) [33–35] in rabbit and in pig, or 24-h infusion of recombinant TFPI in pig [36], immediately prior to angioplasty, also significantly inhibited arterial NH development at 4 weeks. A single bolus of recombinant tick anticoagulant protein (TAP), a factor Xa inhibitor, at time of coronary artery angioplasty followed by a 60-hour intravenous infusion of TAP significantly inhibited NH development assessed at 4 weeks in pigs [37]. Significant inhibition of thrombus and NH after balloon angioplasty of porcine coronary arteries was observed when animals received a 3-day infusion of recombinant TFPI and aspirin compared to infusion with heparin and aspirin [38]. In a rat carotid artery angioplasty model, Wun et al also observed that bolus injections of ANV-6L15 at the time of balloon injury effectively reduced NH at 28-day post-angioplasty [unpublished results]. Thus, short-term administration of the above coagulation inhibitors, by local treatment or intravenous infusion, appeared to be effective in inhibiting not only acute thrombosis but also late NH/stenosis in one-time injury models.

The anti-thrombotic fusion proteins inhibited NH to the same extent as heparin. Heparin is required to prevent perioperative clotting during the AVG placement surgery in swine. Thus, the effects of heparin on NH development alone cannot be determined, due to the confounding issue of clotting. As the NH development was similar in the group treated with the novel anti-thrombotic fusion proteins and that treated with heparin, this suggests that perioperative inhibition of thrombin and its sequela may be insufficient to significantly inhibit AVG stenosis. Although adverse bleeding events were not observed in any treatment group, the novel fusion proteins may have benefit over heparin in other models or in humans because their activity is targeted to the injured vascular surface which may allow for greater or repeat dosing without causing adverse bleeding.

In contrast to wire injury or balloon angioplasty, the AVG experiences continuous insults as the presence of the synthetic graft material stimulates a prolonged, robust foreign-body response that results in the release of inflammatory cytokines and growth factors that promote cell proliferation [23, 39, 40]. In addition, the shunting of high-velocity arterial flow to the low-resistance vein in AVG produces sustained, highly disturbed wall shear stress and flow patterns which also stimulate wall thickening [41]. These multi-factorial insults are far more complex than the one-time insults discussed above and may explain the failure of the anti-thrombotic fusion proteins and of heparin to completely inhibit NH in the AVG setting.

We have previously reported significantly decreased NH development in porcine and canine models of AVG stenosis with repeat percutaneous delivery of sirolimus-laden and paclitaxel-laden polymer gels to the perivascular region of AVG [21, 42]. These small-molecule drugs could be applied perivascularly and were shown to diffuse into the vessel wall. The fusion proteins target the coagulation cascade triggered in the vessel lumen and are too large to diffuse through the vessel wall if applied perivascularly. Whether repeat intravascular administration of the anti-thrombotic fusion proteins is effective in inhibiting NH formation in the AVG setting was not assessed in the present study.

In a pig model of carotid stenosis induced by angioplasty, significantly less NH occurred at 4 weeks after placement of PTFE stent grafts coated with thrombomodulin, an endogenous anticoagulant, compared to uncoated stent grafts [43]. Further, in a randomized clinical trial, commercially available heparin-bonded PTFE grafts were associated with better 1-year patency rates in lower extremity bypass grafts [44]. Thus, coating the PTFE graft with the anti-thrombotic fusion proteins might be efficacious in inhibiting AVG stenosis. However, a retrospective study including data from 223 patients reported that AVG access patency rates were not improved with the heparin-bonded PTFE grafts [45]. Further work is needed on the development of novel, locally delivered, sustained release therapeutics that target thrombosis and/or cell proliferation to improve AVG utility.

## Supporting Information

S1 File Point of care clotting times. Whole blood clotting times for blood from control, TAP-ANV and ANV-6L15-treated pigs.
(XLSX)

S2 File Plasma coagulation results. PT and PTT plasma coagulation results from control, TAP-ANV- and ANV-6L15-treated pigs.
(XLSX)

S3 File Serum chemistry results. ALT, creatinine and WBC results from control, TAP-ANV- and ANV-6L15 treated pigs.
(XLSX)

S4 File MR image analysis results. Effect of anti-thrombotic fusion protein treatment on open lumen area determined by MR imaging.
(XLSX)

S5 File Hyperplasia-to-graft ratio results. Effect of anti-thrombotic fusion protein treatment on hyperplasia-to-graft (H/G) ratios determined by histomorphometry.
(XLSX)
